# O.5.2-2 The relationship between moderate-vigorous physical activity and menopausal symptoms

**DOI:** 10.1093/eurpub/ckad133.243

**Published:** 2023-09-11

**Authors:** Claire Fitzsimons, Janis Reid, Divya Sivaramakrishnan, Nanette Mutrie, Tessa Strain, Ailsa Niven

**Affiliations:** Physical Activity for Health Research Centre, The University of Edinburgh, UK; claire.fitzsimons@ed.ac.uk; Physical Activity for Health Research Centre, The University of Edinburgh, UK; claire.fitzsimons@ed.ac.uk; Physical Activity for Health Research Centre, The University of Edinburgh, UK; claire.fitzsimons@ed.ac.uk; Physical Activity for Health Research Centre, The University of Edinburgh, UK; claire.fitzsimons@ed.ac.uk; Physical Activity for Health Research Centre, The University of Edinburgh, UK; claire.fitzsimons@ed.ac.uk; Physical Activity for Health Research Centre, The University of Edinburgh, UK; claire.fitzsimons@ed.ac.uk

## Abstract

**Purpose:**

There is growing evidence for the benefits of physical activity (PA) during the menopause lifestage (MLS), but limited understanding of women’s experiences of being PA at this time. This study aimed to understand how PA is related to menopausal symptoms during the MLS by: i) assessing the proportion of participants meeting the moderate-vigorous PA (MVPA) guidelines, and perceived PA changes during MLS; ii) assessing prevalence of menopausal symptoms; iii) examining participants’ perceptions of how menopausal symptoms impact PA; iv) investigating the relationship between experiencing symptoms and meeting PA recommendations.

**Methods:**

Participants completed an online survey (Oct-Nov22, n = 655; average age 49.9 years; 56% peri-menopausal; 20% early-post menopause; 24% mid post-menopause) including questions related to meeting MVPA recommendations, experience of 14 menopausal symptoms, and perceptions of symptom impact on likelihood of PA engagement. Descriptive statistics were presented and Chi-squared tests used to examine associations between symptom experience and MVPA guideline compliance, with Bonferroni correction for multiple comparisons.

**Results:**

Seventy-five percent of participants reported achieving MVPA guidelines (150 minutes/week). Fifty-seven percent reported PA levels had decreased during the current menopause life stage. Twelve out of 14 symptoms were experienced by > 50% participants, with changes in mood and brain fog most common (>80%). For 10 out of the 14 symptoms, >50% participants indicated a negative impact on likelihood to engage in PA, with changes to mood, difficulty sleeping (not due to night sweats), and muscle aches and joint pains most detrimental. Whilst not significant, for 10 of the 14 symptoms the proportion meeting the MVPA guidelines was lower amongst those who experienced the symptoms.

**Conclusions:**

Participants had similar levels of MVPA to national norms, although the majority perceived their PA levels had declined. Participants experienced a range of symptoms which were perceived to negatively impact their PA levels, although not statistically evident. The MLS represents a time when being active can be difficult so it is important to find ways to better support women.

**Support/Funding Source:**

This work was funded by SAMH, the Scottish Association for Mental Health.

